# Supplementary material for: In utero tobacco smoke exposure, DNA methylation, and asthma in Latino children
Source: Environ Epidemiol. 2019 Apr 29;3(3):e048. doi: 10.1097/EE9.0000000000000048 (PMC6571182; doi:10.1097/EE9.0000000000000048)
Supplement: Supplementary file 1 [file ee9-3-e048-s001.docx]

**Original Research Article**

***In utero* tobacco smoke exposure, DNA methylation and asthma in Latino children.**

Andreas M. Neophytou;^a,b^ Sam S. Oh;^c^ Donglei Hu;^c^ ; Scott Huntsman;^c^ Celeste Eng;^c^ José R. Rodríguez-Santana;^d^ Rajesh Kumar;^e, f^ John R. Balmes;^a, c^ Ellen A. Eisen;^a^ Esteban G. Burchard.^c^

^a^Division of Environmental Health Sciences, School of Public Health, University of California, Berkeley, Berkeley CA; ^b^Department of Environmental and Radiological Health Sciences, Colorado State University; ^c^Department of Medicine, University of California, San Francisco, San Francisco, CA; ^d^Centro de Neumología Pediátrica, San Juan, Puerto Rico; ^e^Division of Allergy and Immunology, Feinberg School of Medicine, Northwestern University, Chicago, IL; ^f^Ann & Robert H. Lurie Children’s Hospital of Chicago, Chicago, IL.

**Corresponding Author:** Andreas M. Neophytou, ScD, Department of Environmental and Radiological Health Sciences, Colorado State University, 1681 Campus Delivery, Fort Collins, CO, 80523-1681, USA. Email=Andreas.Neophytou@colostate.edu

**Running Head:** *In utero* tobacco smoke, DNA methylation, and asthma

**Conflicts of Interest:** The authors have no conflicts of interest relevant to this article to disclose.

**Funding Support:** Sandler Family Foundation, the American Asthma Foundation, the RWJF Amos Medical Faculty Development Program, Harry Wm. and Diana V. Hind Distinguished Professor in Pharmaceutical Sciences II, National Institutes of Health R01HL117004, R01HL128439, R01HL135156, 1X01HL134589, R01HL141992, National Institute of Health and Environmental Health Sciences R01ES015794, R21ES24844, K99ES027511, the National Institute on Minority Health and Health Disparities P60MD006902 RL5GM118984, R01MD010443 and the Tobacco-Related Disease Research Program under Award Number 24RT-0025, 27IR-0030.

**Data Availability:** GALA II methylation data are publicly available in the Gene Expression Omnibus (GEO) database (accession number GSE77716). For questions regarding code please contact the corresponding author.

**ABSTRACT**

**Background:** Maternal smoking during pregnancy is a risk factor for chronic disease later in life and has been associated with variability of DNA methylation at specific CpG loci. We assessed the role of DNA methylation as a potential mediator of adverse effects of *in utero* tobacco smoke exposures on asthma outcomes in Latino children from the US mainland and Puerto Rico.

**Methods:** Relationships between self-reported exposure and DNA methylation at CpG loci previously reported to be associated with maternal smoking were assessed in a subsample consisting of 572 children aged 8-21 years (310 cases with asthma, 262 healthy controls), sampled from a larger asthma case-control study. Subsequently, we assessed associations between top loci and asthma related outcomes, followed by mediation analysis for loci for which associations with outcomes were observed.

**Results:** Self-reported maternal smoking was associated with a -1.5 (95% confidence interval (CI): -2.4, -0.6) percent lower methylation at CpG locus cg05575921 on the *AHRR* gene; a 1% increase in DNA methylation at the same locus resulted in an odds ratio (OR) of 0.90 (95% CI: 0.83 – 0.96) for the odds of asthma. The OR for the indirect effect of maternal smoking on asthma mediated through methylation at the cg05575921 locus was 1.18 (95% CI: 1.07 – 1.68), compared to the OR for the total effect of exposure in the parent study of 1.48 (95% CI: 1.03 – 2.11).

**Conclusions:** Our findings suggest potential mediation by DNA methylation in the association between maternal smoking during pregnancy and asthma status.

**What this study adds**

In this study we assess the relationship between maternal smoking during pregnancy and DNA methylation in minority children with asthma, as well as consider the role of DNA methylation as a potential mediator for effects of *in utero* tobacco smoke exposures on asthma. We quantify indirect effects of exposure on odds of asthma with respect to DNA methylation at an identified gene in the framework of mediation analysis. Our findings highlight the potential role of DNA methylation as a mediator of the effects of *in utero* exposures on asthma, and further underscore the importance of smoking prevention and cessation.

**INTRODUCTION**

Harmful exposures *in utero* are thought to contribute to the development of chronic disease later in life, including asthma.^1–3^ Maternal tobacco smoking during pregnancy is one of these exposures, with children exposed while *in utero* being at greater risk of adverse health effects,^4–7^ including asthma.^8–10^ Among children with asthma, those with *in utero* tobacco smoke exposures have been shown to have more severe and difficult to control asthma,^11^ while *in utero* tobacco smoke exposure is also adversely related to fetal lung growth^12–14^ and lung function in childhood.^8,14–17^

There is mounting evidence that exposures such as maternal smoking during pregnancy can have epigenetic effects on DNA methylation measured at birth,^18,19^ childhood^18^ and adulthood,^20^ suggesting potentially persisting effects on DNA methylation. DNA methylation is well known to alter gene expression and may be one of the pathways by which *in utero* tobacco smoke exposures exert health effects, with recent studies suggesting that the effects of maternal smoking during pregnancy on birth weight may be mediated through DNA methylation.^21,22^ Fetal lung and placental methylation has also been linked to tobacco smoke exposure during pregnancy, suggesting that DNA methylation may be an intermediate between smoke exposure and adverse effects on lung development.^23^

Maternal smoking during pregnancy remains an important risk factor, with an estimated 12.3% of pregnant women smoking in US, despite increased awareness of the associated risks in recent years.^24^ Minorities tend to have higher prevalence of exposure, with African American and Puerto Rican women more likely to smoke during pregnancy than Whites in the US.^11,24^ Minority children are also disproportionately affected by asthma, with asthma morbidity and mortality being highest among African Americans and Puerto Ricans but lowest in Mexicans.^25,26^ Recent studies report potential effect modification by maternal folate intake,^27^ and normalization of DNA methylation levels in pregnant smokers with Vitamin C treatment, with restoration of methylation at specific CPGs that were also associated with phenotypic respiratory outcomes.^28^ This finding suggests that DNA methylation can be a potential intervention site for the effects of maternal smoking during pregnancy.^29^

In the present study we examine the relationships between DNA methylation at specific cytosine-phosphate-guanine (CpG) loci previously identified to be associated with *in utero* tobacco smoke exposures in a meta-analysis of older pediatric and adolescent cohorts,^18^ with (1) self-reported maternal smoking during pregnancy and with (2) asthma-related outcomes as well as lung function in a study of Latino children from the US mainland and Puerto Rico. We also perform mediation analysis for the effects of *in utero* tobacco smoke exposure on lung function as potentially mediated through DNA methylation loci, in an effort to quantify indirect effects of maternal smoking during pregnancy on asthma, which in theory could be eliminated by intervening on the mediator.

**METHODS**

**Study participants**

This study is based on the Genes-environments and Admixture in Latino Americans (GALA II) study. GALA II is described in detail elsewhere.^11^ Briefly, GALA II is an asthma case-control study of 4702 children (2374 participants with asthma and 2328 healthy controls) recruited from five centers (Chicago, IL; Bronx, NY; Houston, TX; San Francisco Bay Area, CA; and Puerto Rico). Participants were 8–21 years old, self-identified as Latino and must have had four Latino grandparents. Exclusion criteria were: 10 or more pack-years of smoking; any smoking within one year of recruitment date; history of lung diseases other than asthma (cases) or chronic illness (cases and controls); or pregnancy in the third trimester.

**Exposure and methylation data**

Data on self-reported maternal smoking during pregnancy were collected through questionnaires administered in-person with the children’s parents/caretakers by trained bilingual (English–Spanish) interviewers at the time of study recruitment. Specifically, we asked if the child’s mother smoked while she was pregnant with the child (yes/no). Genomic DNA (gDNA) was extracted from whole blood, collected at the time of recruitment using Wizard® Genomic DNA Purification Kits (Promega, Fitchburg, WI). A subset of 572 participants (310 cases with asthma and 262 healthy controls) was measured for DNA methylation using the Infinium Human-Methylation450 BeadChip (Illumina, Inc., San Diego, CA) following the manufacturer’s instructions. Raw genome-wide methylation data were loaded in the R package minfi and assessed for basic quality control metrics, including determination of poorly performing probes with insignificant detection p values above background control probes (i.e., detection p value >0.01). Probes with a single nucleotide polymorphism in the single base extension site were excluded. X and Y chromosomes were removed from the raw methylation values. A total of 321,509 methylation loci were available for analysis. Batch (microarray chip) effect was corrected for using the ComBat function in the R package SVA (surrogate variable analysis) and SWAN normalization was performed to correct for intra-array differences between Illumina Type I and Type II probes. A total of 569 samples passed quality control metrics and were further considered for statistical analyses. Methylation beta values (ranging from 0 to 1) representing the ratio of the intensity of the methylated allele to the sum of the intensities of the methylated and unmethylated alleles were used in analyses. The exposed (self-reported maternal smoking during pregnancy) were oversampled in the subset and latter analysis with asthma status as the outcome in the subset with methylation data is weighted for the probability of selection in the subset conditional on exposure.

**Outcomes and covariates**

Children with asthma (cases) were defined as participants with a history of physician diagnosed asthma and the presence of two or more symptoms of coughing, wheezing, or shortness of breath in the two years preceding enrollment. Among the cases, asthma control was classified according to National Heart, Lung, and Blood Institute measures.^30^ Asthma control was classified as having controlled, partially controlled, or uncontrolled asthma. Asthma and asthma related outcomes were all ascertained at the time of recruitment in the study.

Pulmonary function results in the cases were obtained through spirometry using KoKo PFT Spirometers (nSpire Health Inc., Louisville, CO) according to American Thoracic Society criteria.^31^ Participants were asked not to use their bronchodilator medication 8 hours before spirometry testing. Up to eight tracings were obtained to collect five reproducible expiratory flow–volume curves with less than 5% variability in forced expiratory volume in one second (FEV­_1_ in liters). For analysis, the loop with the best sum of FEV_1_ and forced vital capacity (FVC) was extracted. Information on age, sex, and height was recorded while further demographic information, medical histories, and environmental exposures, were obtained through the questionnaires. Exposure to ambient particulate matter less than 10 μm in diameter (PM_10_) was estimated based on self-reported residential histories from birth (geographic coordinates for each residence were using TomTom/Tele Atlas EZ-Locate software (TomTom, Amsterdam, The Netherlands)) and regional ambient air quality monitoring data acquired from the U.S. Environmental Protection Agency Air Quality System. Exposure was estimated by calculating the inverse distance-squared weighted average from the four closest air quality monitoring stations within 50 km of the residence for each year.

Covariates of interest in our analyses were, sex, age, ethnicity, recruitment region, health insurance, maternal education, self-reported exposures to mold and pets in the household, and average PM­_10­_ exposure in the first 3 years of life. Analyses were conducted in 506 participants with complete covariate data.

**Statistical Analyses**

We assessed relationships between self-reported exposures and methylation values of 148 loci reported with false discovery rate (FDR) adjusted p-values <0.05 in a meta-analysis consisting of eight different older child cohorts as reported in Joubert *et al*.^18^ Linear regression models were used to assess associations between self-reported maternal smoking and DNA methylation levels at specific CpG loci. We accounted for multiple comparisons by controlling the FDR at 5%. In order to account for the case-control design of the study, these models were weighted for the prevalence of the asthma in the target population and proportion of asthma cases in our sample, to obtain consistent estimators for the parameters.^32,33^

We then examined loci with associations with self-reported exposure with FDR adjusted p-values<0.05 in our own sample, as potential mediators of effects of *in utero* tobacco smoke exposure on asthma status, asthma control, as well as pulmonary function. Associations with asthma status were assessed with logistic regression; logistic regression models restricted to the cases were used for asthma control as the outcome with ‘controlled’ asthma as the referent category compared to ‘not well controlled’ or ‘very poorly controlled’ in a single collapsed category. Linear models restricted to the cases were used for lung function parameters, specifically FEV­­_1_, FVC and the FEV_1_/FVC ratio.

Mediation analysis was performed for those CpG loci where 95% confidence intervals (CI) for associations observed for the outcomes of interest did not include the null. For mediation analysis, we also considered additional loci on identified genes that were not on the original a-priori list if CIs for the associations between methylation at the locus and the exposure and the outcome did not include the null. Loci that were individually associated with the exposure and outcome were simultaneously entered in a model for the outcome (adjusted for covariates and the exposure) and those that remained significantly associated with the outcome were retained for mediation analysis. Total effects were partitioned to direct and indirect effects, estimated according to an extension of product method suitable for case-control-data^33,34^. Direct effects (not mediated through methylation) were estimated from the parameter for the exposure (self-reported maternal smoking) in models for the outcome including both exposure and mediators (DNA methylation levels at identified loci) as predictors. Indirect effects (mediated through methylation) were estimated from the product of the parameters for the exposure from models for the mediators and the parameter from the mediators from the model for the outcome, with 95% CIs for direct and indirect effects estimated from 1000 bootstrap samples.^35^ The direct effect here has the interpretation of the contrast in the outcome (e.g., asthma status) between the exposed (to maternal smoking) and the unexposed had the mediator(s) (DNA methylation) was at the levels of the unexposed in everyone. The indirect effect is the contrast in the outcome among the exposed in those where the mediators assume the levels under exposure and those where the mediators assume the levels under no exposure.^34,36^

The exposure of interest was entered in all models as an indicator variable for self-reported maternal smoking during pregnancy. All models further included variables for age, sex, recruitment region and ethnicity, a categorical variable for maternal education (elementary school or less, some high school, high school or equivalent, at least some college, college grad), indicator variable for whether or not the family had health insurance, average PM_10_ exposure in the child’s first three years of life and indicators for parental self-reported exposures to mold and pets in the household ; models for lung function also controlled for height and height squared. Analyses for outcome and methylation associations were controlled for cell type heterogeneity using variables derived from ReFACTor^37^, a method based on principal component analysis designed for cell type heterogeneity correction in epigenetics studies. Sensitivity analyses was performed excluding subjects identified as outliers in ReFACTor (n=13). Data on cell type heterogeneity data in this sample are also extensively detailed by Rahmani et al.^37^

This study was approved by the institutional review board at UC San Francisco. All subjects (or their parents) provided written informed consent.

**RESULTS**

Demographic and asthma outcomes information in the study sample with DNA methylation data is summarized in Table 1. Supplemental Table S1, http://links.lww.com/EE/A40 compares these characteristics between the study sample with DNA methylation data and the entire GALA II study.

Table 2 summarizes the top associations (i.e., FDR adjusted p-value ≤ 0.05 for the current study) out of the 148 loci tested for association with self-reported maternal smoking during pregnancy, sorted by FDR adjusted p-value. Observed distributions for DNA methylation levels for these loci are summarized in boxplots in Supplemental Figure S1, http://links.lww.com/EE/A41. Summary statistics on methylation levels are also in supplemental Table S2, http://links.lww.com/EE/A40. Overall, the range of methylation for each CpG locus was not very wide, with interquartile ranges varying from 3.9% to 14.2%.

Associations between self-reported maternal smoking, as well as methylation at CpG loci and asthma status and asthma control are presented in Table 3. DNA methylation at CpG locus cg05575921 on the *AHRR* gene resulted in the strongest association with asthma status in magnitude, with a 1% increase in methylation levels corresponding to an odds ratio (OR) of 0.90 (95% CI: 0.85 – 0.96). Associations between the same locus and asthma control were in the same direction though weaker and CIs included the null, with a 1% increase in methylation corresponding to an OR of 0.97 (95% CI: 0.89 – 1.06) for asthma control. No other loci considered yielded associations with odds of asthma where the 95% CIs did not include the null. Although asthma control was strongly associated with self-reported maternal smoking, CIs for the associations between this outcome and all the selected loci included the null.

The OR for the total effect of *in utero* tobacco smoke exposures on asthma status using the full GALA II study sample with complete data on self-reported maternal smoking and covariates as listed above (n=3,364) was 1.48 (95% CI: 1.03 – 2.11). In analysis for the same association, in the sub-sample with DNA methylation data (using weights to account for the probability of selection into the sample with DNA methylation data given exposure) the effect estimate was nearly the same, but CIs were much wider and included the null (OR: 1.47 (95% CI: 0.60 – 3.59)).

Results from mediation analysis considering CpG locus cg05575921 as a potential mediator of the effect of maternal smoking during pregnancy are summarized in Table 4. The corresponding direct effect of *in utero* tobacco smoke exposures was an OR of 1.23 (95% CI: 0.48 – 3.16) with an indirect effect mediated through DNA methylation at *AHRR* locus cg05575921 of 1.18 (95% CI: 1.07 – 1.68).

There was a 0.03 decrease (95% CI: -0.05, -0.01) in the FEV­_1_/FVC ratio among the cases associated with self-reported maternal smoking, but 95% CI for individual associations with FEV­_1_ and FVC included the null. Observed associations between lung function parameters and DNA methylation at the CpG loci considered were small in magnitude and generally 95% CIs included the null for both FEV­_1_ and FVC (supplemental figures S2 and S3; <http://links.lww.com/EE/A42> and http://links.lww.com/EE/A43).

**DISCUSSION**

We examined the relationship between DNA methylation at loci previously associated with *in utero* tobacco smoke exposures and asthma-related outcomes in a sample of Latino children from the mainland US and Puerto Rico. We observed associations between DNA methylation levels at the cg05575921 CpG locus on the *AHRR* gene and asthma status. Overall, the observed findings support DNA methylation as a potential biologic pathway of the effects of *in utero* tobacco smoke exposure on asthma status, while evidence for potential effects of DNA methylation at loci considered in this study on asthma control and lung function parameters (FEV_1_, FVC and the FEV_1_/FVC ratio) among children with asthma was weaker and 95% CIs always included the null.

Tobacco smoke exposures have been shown to adversely affect lung growth in children, including effects of *in utero* exposures independent of subsequent postnatal SHS exposures.^9,11^ This suggests potential damage may begin during critical periods of fetal development and is in accordance with the hypothesis that risk of chronic disease begins *in utero.* Tobacco smoke represents a mixture of chemicals, such as nicotine, carbon monoxide, and polycyclic aromatic hydrocarbons, which are known to have harmful effects. Nicotine has been linked with differentiation of developing lung cells into abnormal phenotypes,^38^ and has been shown to cross the placenta *in utero*.^14,39^

Epigenetic regulation as a potential mechanism of disease is an area of particular interest in recent asthma literature^40^ and also has been strongly linked with *in utero* tobacco smoke exposures. Gene expression can be modulated by epigenetic modifications, including methylation of CpG islands in specific genes, thus affecting fetal development and contributing to chronic disease. Epigenetic modifications in relation to *in utero* tobacco smoke exposures have been reported in newborns and placental and cord blood samples^18,22^ but also later in childhood^18^ and adulthood,^20^ indicating potentially persistent changes in the epigenome associated with these exposures. The harmful effects of *in utero* tobacco smoke exposures are also documented throughout the life course, with potential damage beginning during fetal development, resulting in attenuated lung function and increased risk of childhood asthma^9,41^ early in life but also persisting effects later in childhood^16,42^ and potentially adulthood.^15^ DNA methylation can potentially be intervened upon to reduce effects of harmful exposures mediated through these mechanisms as suggested by a recent study indicating restoration of DNA methylation in pregnant smokers through Vitamin C treatment.^28,29^ Dietary interventions have also been shown to potentially ameliorate health effects of air pollution exposures mediated through DNA methylation.^43^ This could prove to be a useful mechanism to supplement interventions targeting a reduction in the prevalence of exposure, if interventions on the mediator can help reduce exposure effects after the exposure has already taken place (i.e. potential dietary supplementation in children born to mothers who smoked).

Our results are consistent with the hypothesis that DNA methylation is a potential biologic mediator of the harmful effects of *in utero* tobacco smoke exposures on asthma and asthma-related outcomes, particularly with respect to methylation at the *AHRR* gene. Differential methylation at *AHRR* loci has been reported in other studies of maternal smoking^18,19^ where exposure results in reduced methylation. The aryl hydrocarbon receptor has a role in detoxification and immune system regulation^44^ and has been linked with inflammation in the lung.^45^ Epigenetic changes in *AHRR* have been shown to persist during childhood.^14,46^ Epigenetic changes at the *AHRR* gene have also been observed in studies of adolescent smokers.^47^.In our study, self-reported maternal smoking was associated with lower methylation at an *AHRR* CpG locus (cg05575921). This locus has consistently been reported to be differentially methylated in association to smoking exposures, including in studies using maternal plasma cotinine, a sensitive tobacco smoke exposure biomarker, to assess exposure.^18,19,21,47,48^ In turn, an increase in methylation at this locus was associated with decreased risk of asthma. In our study population, the same directions of associations (though weaker in magnitude and with 95 CIs including the null) were observed for asthma control, FEV_1_ and FEV_1_/FVC among children and adolescents with asthma. Results for asthma control and lung function parameters were limited to the asthma cases, therefore were less powered.

Of the loci considered, differential methylation associated with self-reported maternal smoking at the *AHRR* cg05575921 locus was the only locus consistently associated with harmful effects in all outcomes considered, though confidence intervals often included the null. *AHRR* loci, however, were not found among the top loci associated with childhood asthma in an epigenome-wide meta-analysis of DNA methylation and childhood asthma.^49^ Our approach had exposure-mediator associations as the starting point, whereas analyses of epigenome-wide associations jointly for exposures and outcomes of interest could be a future direction.

Results from mediation analysis are subject to several assumptions, so interpretation of these findings should be made in light of these assumptions as well as other limitations of observational data. In addition to the assumptions of no unmeasured exposure-outcome confounders required for analyses of total effects, assumptions of no unmeasured exposure-mediator, or mediator-outcome confounders and no mediator-outcome confounders affected by the exposure are also required. Our results are also estimated under the assumption of no exposure-mediator interactions. The assumption of no mediator-outcome confounding will likely be particularly problematic in studies examining DNA methylation as a potential mediator, as factors such as maternal diet and genetics could all act as mediator-outcome confounders. DNA methylation at other loci could also be a confounder even if unassociated with exposure and could be an issue given the loci reported to be associated with the outcome as highlighted by Reese et al.^49^ Failure of this assumption may have resulted in overestimation of indirect effects in our case.

A further point of caution in mediation analysis is bias due to potential exposure misclassification. While non-differential misclassification of exposure is generally expected to lead to bias towards the null with respect to total effects, bias in indirect effects can be in either direction.^34^ Mother’s reported smoking during pregnancy is subject to misclassification, but is generally considered a valid measure of fetal tobacco smoke exposure.^50^ In our study self-reported exposures are consistent with previous reports and national estimates by race/ethnicity^11,51^, so misclassification of exposure is not expected to be a major issue. The exposure-mediator associations seen in our study were also observed in studies that used cotinine to assess exposure. Methylation differences between the exposed and unexposed based on self-reported measures in our study were in the same direction for 25 out of 26 CpG loci reported in a study using mid-pregnancy maternal cotinine to determine *in utero* tobacco smoke exposures.^52^ This lends further confidence to our self-reported measure of exposure.

The cross-sectional nature of the data is a limitation of the study. Blood samples were collected at one point in time, concurrent with outcome assessment, which was retrospective with respect to ascertainment of asthma status and asthma-related outcomes. The temporal relationship between DNA methylation and asthma outcomes cannot therefore be established with certainty in this dataset. In this regard DNA methylation measured in cord blood would be more advantageous; however, evidence in the literature supports effects of *in utero* exposures on DNA methylation, observed early in life and prior to manifestation of chronic disease and other clinical outcomes, and therefore can be considered as potential intermediates. Furthermore DNA methylation associations from prenatal exposures such as parental smoke exposure could persist later in life^20,53^ and DNA methylation measured in child blood may be still be a useful marker for mediators.

Among the strengths of the study was the ability to adjust for potential confounders and co-exposures such as socioeconomic status variables and other co-exposures. Our study is also based on a sample of Latino children from the GALA II study, the largest gene-environment study of pediatric asthma in Latinos in the US. Some minority children, particularly Puerto Ricans, are both more at risk for asthma-related morbidity and mortality,^25^ and have higher prevalence of maternal smoking during pregnancy compared to the rate for the entire US population. Our target population therefore, is of particular clinical and public health interest given the potential differences in susceptibility to the outcome in light of the greater burden of exposure. In addition, a previous study in this population indicated that methylation at loci associated with environmental exposures including maternal smoking was also associated with ethnicity and genetic ancestry.^54^ This finding in addition to the differential prevalence of exposure and outcome by race/ethnicity introduces the potential for lack of transportability of effects from one population to another and it is therefore important to assess these associations in populations of different racial/ethnic backgrounds. Lastly, and to our knowledge, the current study is the first to apply mediation analysis of *in utero* tobacco smoke exposure effects on asthma, mediated though epigenetic modification of DNA methylation. DNA methylation as a potential mediator of effects is an area of increasing interest in asthma epidemiology, and one which may provide insights as to the biochemical mechanisms through which external exposures exert their effects.^40^

In summary, we observed associations between DNA methylation at loci previously linked to *in utero* tobacco smoke exposure, and asthma related outcomes. Our findings are consistent with DNA methylation acting as a mediator of potential effects of *in utero* tobacco smoke exposures on asthma status. Potential future studies including mediation analysis in longitudinal prospective studies where exposure, mediators and outcomes are assessed in a temporal order as hypothesized, will benefit our overall understanding of *in utero* tobacco smoke exposure, as will studies with gene expression as an additional part of the pathway of mediated mechanisms of disease.

**REFERENCES**

1. Barker DJ, Osmond C, Golding J, Kuh D, Wadsworth ME. Growth in utero, blood pressure in childhood and adult life, and mortality from cardiovascular disease. *BMJ*. 1989;298(6673):564-567.

2. Waterland RA, Michels KB. Epigenetic epidemiology of the developmental origins hypothesis. *Annu Rev Nutr*. 2007;27:363-388.

3. Stocks J, Hislop A, Sonnappa S. Early lung development: lifelong effect on respiratory health and disease. *Lancet Respir Med*. 2013;1(9):728-742.

4. US Department of Health and Human Services. *The Health Consequences of Smoking — 50 Years of Progress A Report of the Surgeon General (US Department of Health and Human Services, Centers for Disease Control and Prevention, National Center for Chronic Disease Prevention and Health Promotion).* Atlanta, GA; 2014.

5. DiFranza JR, Aligne CA, Weitzman M. Prenatal and Postnatal Environmental Tobacco Smoke Exposure and Children’s Health. *Pediatrics*. 2004;113(4):1007-1015.

6. Best D. Secondhand and Prenatal Tobacco Smoke Exposure. *Pediatrics*. 2009;124(5):e1017-e1044.

7. US Department of Health and Human Services. *The Health Consequences of Involuntary Exposure to Tobacco Smoke: A Report of the Surgeon General.* Washington, DC; 2006.

8. Stick SM, Burton PR, Gurrin L, Sly PD, LeSouëf PN. Effects of maternal smoking during pregnancy and a family history of asthma on respiratory function in newborn infants. *Lancet*. 1996;348(9034):1060-1064.

9. Gilliland FD, Li YF, Peters JM. Effects of maternal smoking during pregnancy and environmental tobacco smoke on asthma and wheezing in children. *Am J Respir Crit Care Med*. 2001;163(2):429-436.

10. Hu FB, Persky V, Flay BR, Zelli A, Cooksey J, Richardson J. Prevalence of asthma and wheezing in public schoolchildren: association with maternal smoking during pregnancy. *Ann Allergy Asthma Immunol*. 1997;79(1):80-84.

11. Oh SS, Tcheurekdjian H, Roth LA, et al. Effect of secondhand smoke on asthma control among black and Latino children. *J Allergy Clin Immunol*. 2012;129(6):1478-1483.e7.

12. Wongtrakool C, Roser-Page S, Rivera HN, Roman J. Nicotine alters lung branching morphogenesis through the alpha7 nicotinic acetylcholine receptor. *Am J Physiol Lung Cell Mol Physiol*. 2007;293(3):L611-L618.

13. Wu ZX, Hunter DD, Kish VL, Benders KM, Batchelor TP, Dey RD. Prenatal and early, but not late, postnatal exposure of mice to sidestream tobacco smoke increases airway hyperresponsiveness later in life. *Environ Health Perspect*. 2009;117(9):1434-1440.

14. Gibbs K, Collaco JM, McGrath-Morrow SA. Impact of tobacco smoke and nicotine exposure on lung development. *Chest*. 2016;149(2):552-561.

15. Upton MN, Watt GCM, Smith GD, McConnachie A, Hart CL. Permanent effects of maternal smoking on offsprings’ lung function. *Lancet*. 1998;352(9126):453.

16. Gilliland FD, Berhane K, McConnell R, et al. Maternal smoking during pregnancy, environmental tobacco smoke exposure and childhood lung function. *Thorax*. 2000;55(4):271-276.

17. Gilliland FD, Berhane K, Li Y-F, Rappaport EB, Peters JM. Effects of early onset asthma and in utero exposure to maternal smoking on childhood lung function. *Am J Respir Crit Care Med*. 2003;167(6):917-924.

18. Joubert BR, Felix JF, Yousefi P, et al. DNA Methylation in Newborns and Maternal Smoking in Pregnancy : Genome-wide Consortium Meta-analysis. *Am J Hum Genet*. 2016;98(4):680-696.

19. Reese SE, Zhao S, Wu MC, et al. DNA Methylation Score as a Biomarker in Newborns for Sustained Maternal Smoking during Pregnancy. *Environ Health Perspect*. 2017;125(4):760-766.

20. Richmond RC, Suderman M, Langdon R, Relton CL, Davey Smith G. DNA methylation as a marker for prenatal smoke exposure in adults. *Int J Epidemiol*. 2018;47(4):1120-1130.

21. Küpers LK, Xu X, Jankipersadsing SA, et al. DNA methylation mediates the effect of maternal smoking during pregnancy on birthweight of the offspring. *Int J Epidemiol*. 2015;44(4):1224-1237.

22. Morales E, Vilahur N, Salas LA, et al. Genome-wide DNA methylation study in human placenta identifies novel loci associated with maternal smoking during pregnancy. *Int J Epidemiol*. 2016;45(5):1644-1655.

23. Chhabra D, Sharma S, Kho AT, et al. Fetal lung and placental methylation is associated with in utero nicotine exposure. *Epigenetics*. 2014;9(11):1473-1484.

24. Tong VT, Jones JR, Dietz PM, D’Angelo D, Bombard JM, Centers for Disease Control and Prevention (CDC). Trends in smoking before, during, and after pregnancy - Pregnancy Risk Assessment Monitoring System, United States, 40 sites, 2000-2010. *MMWR Surveill Summ*. 2013;62(6):1-19.

25. Akinbami LJ, Moorman JE, Bailey C, et al. Trends in Asthma Prevalence, Health Care Use, and Mortality in the United States, 2001-2010. 2012:1-8.

26. Lara M, Akinbami L, Flores G, Morgenstern H. Heterogeneity of childhood asthma among Hispanic children: Puerto Rican children bear a disproportionate burden. *Pediatrics*. 2006;117(1):43-53.

27. Zhang B, Hong X, Ji H, et al. Maternal smoking during pregnancy and cord blood DNA methylation: new insight on sex differences and effect modification by maternal folate levels. *Epigenetics*. 2018;13(4):505-518.

28. Shorey-Kendrick LE, McEvoy CT, Ferguson B, et al. Vitamin C prevents offspring DNA methylation changes associated with maternal smoking in pregnancy. *Am J Respir Crit Care Med*. 2017;196(6):745-755.

29. Sharma S. The epigenetics of intrauterine smoke exposure: Can maternal Vitamin C supplementation prevent neonatal respiratory disease? *Am J Respir Crit Care Med*. 2017;196(6):672-674.

30. National Asthma Education and Prevention Program (National Heart Lung and Blood Institute). *Expert Panel Report 3: Guidelines for the Diagnosis and Management of Asthma: Full Report 2007.* Bethesda, MD; 2007.

31. American Thoracic Society. Standardization of Spirometry, 1994 Update. American Thoracic Society. *Am J Respir Crit Care Med*. 1995;152(3):1107-1136.

32. van der Laan MJ. Estimation based on case-control designs with known prevalence probability. *Int J Biostat*. 2008;4(1):Article 17.

33. VanderWeele TJ, Vansteelandt S. Odds Ratios for Mediation Analysis for a Dichotomous Outcome. *Am J Epidemiol*. 2010;172(12):1339-1348.

34. Vanderweele TJ. *Explanation in Causal Inference: Methods of Mediation and Interaction*. New York, NY: Oxford University Press; 2015.

35. VanderWeele T, Vansteelandt S. Mediation Analysis with Multiple Mediators. *Epidemiol Method*. 2014;2(1):95-115.

36. Richiardi L, Bellocco R, Zugna D. Mediation analysis in epidemiology: Methods, interpretation and bias. *Int J Epidemiol*. 2013;42(5):1511-1519.

37. Rahmani E, Zaitlen N, Baran Y, et al. Sparse PCA corrects for cell type heterogeneity in epigenome-wide association studies. *Nat Methods*. 2016;13(5):443-445.

38. Rehan VK, Asotra K, Torday JS. The effects of smoking on the developing lung: Insights from a biologic model for lung development, homeostasis, and repair. *Lung*. 2009;187(5):281-289.

39. Jauniaux E, Gulbis B, Acharya G, Thiry P, Rodeck C. Maternal tobacco exposure and cotinine levels in fetal fluids in the first half of pregnancy. *Obstet Gynecol*. 1999;93(1):25-29.

40. Harb H, Renz H. Update on epigenetics in allergic disease. *J Allergy Clin Immunol*. 2015;135(1):15-24.

41. Jaakkola JJK, Gissler M. Maternal Smoking in Pregnancy, Fetal Development, and Childhood Asthma. *Am J Public Health*. 2004;94(1):136-140.

42. Cunningham J, Dockery DW, Speizer FE. Maternal smoking during pregnancy as a predictor of lung function in children. *Am J Epidemiol*. 1994;139(12):1139-1152.

43. Zhong J, Colicino E, Lin X, et al. Cardiac autonomic dysfunction: Particulate air pollution effects are modulated by epigenetic immunoregulation of Toll-like receptor 2 and dietary flavonoid intake. *J Am Heart Assoc*. 2015;4(1):1-20.

44. Hanieh H. Toward understanding the role of aryl hydrocarbon receptor in the immune system: current progress and future trends. *Biomed Res Int*. 2014;2014:520763.

45. Beamer CA, Shepherd DM. Role of the aryl hydrocarbon receptor (AhR) in lung inflammation. *Semin Immunopathol*. 2013;35(6):693-704.

46. Richmond RC, Simpkin AJ, Woodward G, et al. Prenatal exposure to maternal smoking and offspring DNA methylation across the lifecourse: Findings from the Avon Longitudinal Study of Parents and Children (ALSPAC). *Hum Mol Genet*. 2015;24(8):2201-2217.

47. Beach SRH, Lei MK, Ong ML, Brody GH, Dogan M V., Philibert RA. MTHFR methylation moderates the impact of smoking on DNA methylation at AHRR for African American young adults. *Am J Med Genet Part B Neuropsychiatr Genet*. 2017;174(6):608-618.

48. Zeilinger S, Kühnel B, Klopp N, et al. Tobacco Smoking Leads to Extensive Genome-Wide Changes in DNA Methylation. *PLoS One*. 2013;8(5).

49. Reese SE, Xu C-J, Dekker HT Den, et al. Epigenome-wide meta-analysis of DNA methylation and childhood asthma. *J Allergy Clin Immunol*. 2019;[Epub ahea.

50. Pickett KE, Rathouz PJ, Kasza K, Wakschlag LS, Wright R. Self-reported smoking, cotinine levels, and patterns of smoking in pregnancy. *Paediatr Perinat Epidemiol*. 2005;19(5):368-376.

51. Perreira KM, Cortes KE. Race/ethnicity and nativity differences in alcohol and tobacco use during pregnancy. *Am J Public Health*. 2006;96(9):1629-1636.

52. Joubert BR, Håberg SE, Nilsen RM, et al. 450K epigenome-wide scan identifies differential DNA methylation in newborns related to maternal smoking during pregnancy. *Environ Health Perspect*. 2012;120(10):1425-1431. doi:10.1289/ehp.1205412

53. Cardenas A, Rifas-Shiman SL, Agha G, et al. Persistent DNA methylation changes associated with prenatal mercury exposure and cognitive performance during childhood. *Sci Rep*. 2017;7(1):1-14.

54. Galanter JM, Gignoux CR, Oh SS, et al. Differential methylation between ethnic sub-groups reflects the effect of genetic ancestry and environmental exposures. *Elife*. 2017;6:e20532.

Table 1: Demographic characteristics of study sample of 572 Latino children with methylation data from the GALA II asthma case control study.

| Characteristic, n (%) | Cases (N=310) | Controls (N=262) |
| --- | --- | --- |
| Male | 156 (50.3) | 130 (49.6) |
| Age, mean±sd | 12.4±3.4 | 12.7±3.5 |
| Maternal smoking during pregnancy | 59 (19.0) | 21 (8.0) |
| Maternal education^*^ |  |  |
| Less than high school | 104 (33.9) | 121 (46.5) |
| High school grad. or equivalent | 89 (29.0) | 64 (24.6) |
| At least some college | 114 (37.1) | 75 (28.8) |
| Family had Health insurance | 292 (94.2) | 231 (88.1) |
| Mold exposure in the household | 102 (32.9) | 61 (23.3) |
| Pets in the household | 114 (36.8) | 104 (39.7) |
| Recruitment region |  |  |
| Chicago | 91 (29.4) | 85 (32.4) |
| Houston | 21 (6.8) | 28 (10.7) |
| New York | 21 (6.8) | 31 (11.8) |
| Puerto Rico | 132 (42.6) | 60 (22.9) |
| SF Bay Area | 45 (14.5) | 58 (22.1) |
| Ethnicity |  |  |
| Mexican | 124 (40) | 152 (58.0) |
| Other Latino | 40 (12.9) | 37 (14.1) |
| Puerto Rican | 146 (47.1) | 73 (27.9) |
| Asthma control |  |  |
| Controlled | 88 (28.4) | . |
| Partially controlled | 106 (34.2) | . |
| Uncontrolled | 116 (37.4) | . |
| PM_10_ in first 3 years (μg/m^3^ mean±sd) | 29.5±5.7 | 29.2±5.3 |

Abbreviations: GALA II: Genes-environments and Admixture in Latino Americans

^*^Information on maternal education, insurance and PM­­_10_ exposure was available in 567, 567 and 517 subjects respectively.

Table 2: Difference in methylation levels associated with self-reported maternal smoking for selected CpG loci, presented by increasing FDR adjusted p-value.^*^

| CpG locus (gene)^†^ | Change (95% CI), p-value | FDR adjusted p-value |
| --- | --- | --- |
| cg18132363 (*LINC00473*)† | -4.1 (-6.0, -2.1), <0.0001 | 0.0049 |
| cg26764244 (*GNG12*) | -2.3 (-3.4, -.2), <0.0001 | 0.0049 |
| cg13822849 (*OLFM1*) | 1.5 (0.6, 2.5), <0.001 | 0.0400 |
| cg05575921 (*AHRR*) | -1.5 (-2.4, -0.6), 0.002 | 0.0489 |
| cg22549041 (*CYP1A1*) | 3.6 (1.2, 5.9), 0.0019 | 0.0489 |
| cg26889659 (*EXOC2*) | -5.7 (-9.2, -2.1), 0.0020 | 0.0489 |
| cg25189904 (*GNG12*) | -2.6 (-4.3, -0.9), 0.0023 | 0.05 |

Abbreviations: FDR: false discovery rate

^*^Results from linear models with methylation as the outcome and adjusted for age, sex, ethnicity, recruitment region, maternal education, health insurance, PM_10_ exposure in the first 3 years of life, self-reported mold and pets in the household and ReFACTor variables. Models weighted for the prevalence of the outcome and proportion of cases.

^†^The gene for CpG locus cg18132363 is the nearest (within 10Mb) genes to the locus as opposed to a mapped gene.

Table 3: Odds ratios (95% CIs) for asthma status and asthma control associated with a 1% methylation increase at selected loci and self-reported maternal smoking during pregnancy.

| CpG locus  (associated gene)^*^ | OR (95% CI) | |
| --- | --- | --- |
|  | Asthma status^†^  (n=572) | Asthma Control^†^  (n=310) |
| Maternal smoking | 1.47 (0.60 – 3.59)^‡^ | 2.03 (0.88 – 4.72) |
| cg18132363 (*LINC00473*)^*^ | 1.02 (0.98 – 1.05) | 0.98 (0.92 – 1.03) |
| cg26764244 (*GNG12*) | 1.03 (0.97 – 1.09) | 0.97 (0.90 – 1.05) |
| cg13822849 (*OLFM1*) | 1.06 (0.98 – 1.13) | 0.90 (0.82 – 1.00) |
| cg05575921 (*AHRR*) | 0.90 (0.83 – 0.96) | 0.96 (0.87 – 1.05) |
| cg22549041 (*CYP1A1*) | 0.99 (0.97 – 1.02) | 0.99 (0.97 – 1.01) |
| cg26889659 (*EXOC2*) | 1.01 (0.99 – 1.03) | 1.01 (0.98 – 1.04) |
| cg25189904 (*GNG12*) | 1.00 (0.96 – 1.04) | 1.01 (0.95 – 1.07) |

Abbreviations: CI: confidence interval; OR: odds ratio

^*^The gene for CpG locus cg18132363 is the nearest (within 10Mb) gene to the locus as opposed to a mapped gene

^†^Results for asthma status are from logistic models for all subjects with DNA methylation data, while results for asthma control are from logistic models restricted to the cases. All models adjusted for age, sex, ethnicity, recruitment region, maternal education, health insurance, PM_10_ exposure in the first 3 years of life, self-reported mold and pets in the household and and ReFACTor variables.

^‡^The OR corresponding to self-reported maternal smoking is weighted by the probability of selection from the original GALA II study as the exposed were oversampled for the subset in the current study.

Table 4: Associations between DNA methylation at selected *AHRR* loci (mediators) and self-reported maternal smoking (exposure) from a model for the mediator, as well as associations between DNA methylation and asthma status (outcome) from a model for the outcome. Model parameters from both models are used to estimate indirect (mediated) effects of exposure (results based on the GALA II subsample with DNA methylation and complete covariate data (n=506)).

| Mediator model^*^ | Change in DNA methylation (se) |  |
| --- | --- | --- |
| *AHRR* locus cg05575921 | -0.015 (0.005) |  |
|  |  |  |
|  |  |  |
| Outcome model^†^ |  | Direct and Indirect Effects |
|  | Change in log(odds) of asthma (se) | OR (95% CI)^‡^ |
| Maternal smoking | 0..21 (0.48) | DE: 1.23 (0.48 – 3.16) |
| *AHRR* locus cg05575921 | -11.01 (3.47) | IE: 1.18 (1.07 – 1.68) |

Abbreviations: CI: confidence interval ; DE: direct effect; GALA II: GALA II: Genes-environments and Admixture in Latino Americans; IE: indirect effect; OR: odds ratio.

^*^Model for the mediator, and adjusted for age, sex, ethnicity, recruitment region, maternal education, health insurance, PM_10_ exposure in the first 3 years of life, self-reported mold and pets in the household and ReFACTor variables. Models weighted for the prevalence of the outcome and proportion of cases. Standard errors estimated based on bootsraps.

†Model for the outcome was a logistic model including the exposure and mediators and adjusted for age, sex, ethnicity, recruitment region, maternal education, health insurance, PM_10_ exposure in the first 3 years of life, self-reported mold and pets in the household and ReFACTor variables and weighted for the probability of selection in the subsample.

^‡^The log(odds) of the direct effect is equivalent model parameter for the exposure in the outcome model. The log(odds) for the indirect effect are the product between the model parameter for the cpg locus in the outcome model times the model parameter for the exposure in the respective mediator model i.e. the OR for IE: exp((-0.015)*(-11.01)). 95% CIs estimated based on bootstraps.
